# Implementation of Dual-Source RF Excitation in 3 T MR-Scanners Allows for Nearly Identical ADC Values Compared to 1.5 T MR Scanners in the Abdomen

**DOI:** 10.1371/journal.pone.0032613

**Published:** 2012-02-29

**Authors:** Raghuram K. Rao, Philipp Riffel, Mathias Meyer, Paul J. Kettnaker, Andreas Lemke, Stefan Haneder, Stefan O. Schoenberg, Henrik J. Michaely

**Affiliations:** 1 Institute of Clinical Radiology and Nuclear Medicine, University Medical Center Mannheim, Medical Faculty Mannheim, Heidelberg University, Heidelberg, Germany; 2 Department of Radiology, The University of Texas, Medical Branch, Galveston, Texas, United States of America; 3 Department of Computer Assisted Clinical Medicine, University Medical Center Mannheim, Medical Faculty Mannheim, Heidelberg University, Heidelberg, Germany; Institution of Automation - CAS, China

## Abstract

**Background:**

To retrospectively and prospectively compare abdominal apparent diffusion coefficient (ADC) values obtained within in a 1.5 T system and 3 T systems with and without dual-source parallel RF excitation techniques.

**Methodology/Principal Findings:**

After IRB approval, diffusion-weighted (DW) images of the abdomen were obtained on three different MR systems (1.5 T, a first generation 3 T, and a second generation 3 T which incorporates dual-source parallel RF excitation) on 150 patients retrospectively and 19 volunteers (57 examinations total) prospectively. Seven regions of interest (ROI) were throughout the abdomen were selected to measure the ADC. Statistical analysis included independent two-sided t-tests, Mann-Whitney U tests and correlation analysis. In the DW images of the abdomen, mean ADC values were nearly identical with nonsignificant differences when comparing the 1.5 T and second generation 3 T systems in all seven anatomical regions in the patient population and six of the seven in the volunteer population (p>0.05 in all distributions). The strength of correlation measured in the volunteer population between the two scanners in the kidneys ranged from r = 0.64–0.88 and in the remaining regions (besides the spleen), r>0.85. In the patient population the first generation 3 T scanner had different mean ADC values with significant differences (p<0.05) compared to the other two scanners in each of the seven distributions. In the volunteer population, the kidneys shared similar ADC mean values in comparison to the other two scanners with nonsignificant differences.

**Conclusions/Significance:**

A second generation 3 T scanner with dual-source parallel RF excitation provides nearly identical ADC values compared with the 1.5 T imaging system in abdominal imaging.

## Introduction

In recent years, advancements in magnetic resonance imaging (MRI) have allowed for evaluation of pathologic conditions with objective measurements. Commonly referred to as quantitative MR, this imaging approach aims to numerically and graphically reveal biologic, oftentimes microscopic, attributes of tissues, commonly after manipulation of MR-inducible properties. In abdominal imaging, several parameters have already been quantified using such measures, such as proton density, T1/T2/T2* relaxation, magnetic transfer, and more recently diffusion [1]. Of these techniques, diffusion-weighted imaging is particularly gaining rapid popularity in the abdomen and pelvis.

Numerous clinical applications now exist with diffusion-weighted imaging (DWI). The extent of pathophysiological characterizations includes defining organ functions (renal failure), rating disease severity/chronicity (liver cirrhosis, fibrosis, chronic pancreatitis), identifying infection (pyelonephritis, abscesses), assessing the acuity of Crohn's disease, and localizing lymph nodes [1], [2], [3]. However, DWI is receiving most attention for its potential ability to detect and characterize malignant disease. There is even suggestion that DWI may be more sensitive than contrast-enhanced MR sequences in detecting small malignant lesions [4].

The apparent diffusion coefficient (ADC), which is a quantitative MR-biomarker determined by tissue water Brownian motion is affected by tissue perfusion and cellularity, can be calculated from DW sequences. Although numerous recent investigations have correlated a lower ADC value with several malignancies, discrete and reproducible threshold numbers have been difficult to obtain. One of the main limitations in defining such cutoff ADC values is the current lack of technical standardization for imaging parameters, such as the selection of the number and value of b-factors, and MR scanning technologies [5], [6].

Until recently, most studies in the literature evaluating the accuracy and reproducibility of ADC values in the abdomen have been confined to 1.5 T scanners, as DW images obtained with 3 T MR systems have been limited by artifacts caused by B_1_ field inhomogeneity inherent to higher field strengths [7], [8]. Although recent studies have investigated the usage of 3 T DWI in peripheral structures such as the kidneys [9], [10], [11], artifacts and inappropriate fat suppression at this field strength often hamper the ability to obtain dependable ADC values in deeper anatomical distributions such as the left lobe or the caudate lobe of the liver, which are particularly prone to B_1_ field inhomogeneities. Therefore most investigations of the abdomen that analyze more internal regions using DW sequences continue to be restricted to 1.5 T MR scanners. To date, it is still uncertain if the numerous ADC values extrapolated from 1.5 T scanners that characterize normal anatomy and pathologic lesions within the abdomen can be utilized when performing MRI studies on 3 T [12]. New developments in 3 T technologies provide dual-source parallel RF excitation along with independent radiofrequency (RF) shimming. These advancements also aim to reduce the degree of artifact caused by B_1_ inhomogeneity experienced by the first generation 3 T systems. Preliminary results have suggested improved image quality of DWI when comparing the second generation systems to their first generation 3 T counterparts (own submitted data and [13], [14]).

Although there is limited ability to reproduce ADC values from 1.5 T with first generation 3 T systems [12], no studies have compared ADC values between 1.5 T and second generation 3 T scanners. Therefore our study aims to compare ADC values measured in several anatomical regions of the abdomen using 1.5 T, first generation 3 T, and second generation 3 T MR scanners.

## Materials and Methods

### Patients

This study contained two populations—one consisting of 150 patients and the other comprised of 19 volunteers. The institutional review board (IRB name: Medizinische Ethikkomission II der Medizinischen Fakultät Mannheim, Heidelberg Universität; Germany) waived the requirement of informed patient consent in the retrospective patient population, but information gathered on this population was performed in compliance with HIPAA guidelines. The IRB approved the prospective study of the volunteers, who signed a written consent form prior to MR imaging.

The patient population consisted of 150 patients (mean age, 52.2 years ± 18.5 years [standard deviation]; age range, 9–83 years; 79 men and 71 women). The patients were retrospectively selected as the 50 most recent clinical patients being scanned in one of the three investigated scanners (1.5 T versus the first generation 3 T versus the second generation 3 T which implements a dual source RF excitation technique) through July 2011. The only inclusion criterion was limiting the selection of patients to those who had routine protocol DW sequences (as listed below) of the upper abdomen. No exclusion criteria were defined. The second study population of 19 healthy volunteers (mean age, 39.5 years+14.4 years [standard deviation]; age range, 19–62 years; 12 men and 7 women) was prospectively selected and assigned to undergo MR imaging in all of the three above-mentioned scanners. No inclusion criteria were made. The study was limited from volunteers less than 18 years of age and those who had contraindications to MR imaging (incompatible metal implants, cochlear implants, or pacemakers). After the volunteers signed a formal consent form, none were restricted or excluded from the study.

### MR Imaging

Three different MR scanners were used: a 1.5 T MR system (MAGNETOM Avanto 32×76 1.5 T; Siemens Healthcare; Erlangen, Germany), a first generation 3 T MR system (MAGNETOM Tim Trio 32×76 3 T; Siemens), and second generation 3 T MR imaging system with TrueForm magnet design (MAGNETOM Skyra; Siemens). The TrueForm technology represents a basic two-way parallel transmission system characterized by a 90° difference in the phase and amplitude RF excitation of the MR-systems's body coil which allows for a a more homogenous excitation of the volume of interest. All three MRI scanners were equipped with the same gradient systems. The studies were performed with the systems' standard anterior body matrix coils (six independent coil elements in the 1.5 T and first generation 3 T; 18 independent coil elements in the second generation 3 T) and the scanners' included posterior spine matrix coils (with eight coil elements in all three MR-scanners).

In the patient population, ADC values were calculated from the routinely used b-values of 50/400/800 s/mm^2^, and in the volunteer population, the b-values were 0/50/100/200/400/800 s/mm^2^. All images were acquired during free breathing without respiratory triggering. Slice thickness, interslice gap, and spatial resolution remained similar across all three scanners in both populations (Table 1 and Table 2).

**Table 1:** Imaging parameters in the three imaging systems used for the patient population.

	1.5 T	1^st^ gen. 3 T	2^nd^ gen. 3 T
**TR/TE [ms]**	5600/75	6000/76	6400/63
**Sequence type**	EPI-SE	EPI-SE	EPI-SE
**FOV [mm×mm]**	380×308	380×308	380×308
**Matrix**	192×156	192×156	192×156
**Slice thickness [mm]**	6	5	5
**Interslice gap [mm]**	0	0	0
**Spatial resolution [mm^3^]**	2.0×2.0×6.0	2.0×2.0×5.0	2.0×2.0×5.0
**Number slices**	32	33	35
**b-values**	50, 400, 800	50, 400, 800	50, 400, 800
**Parallel imaging**	GRAPPA 2	GRAPPA 2	GRAPPA 2
**Acquisition time [min]**	4:30	5:06	4:46
**Respiratory control**	Free breathing	Free breathing	Free breathing
**Fat suppression**	SPAIR	SPAIR	SPAIR
**Averages**	4	4	3
**Bandwidth [Hz/px]**	1736	1736	1736

**Table 2:** Imaging parameters in the three imaging systems used for the volunteer population.

	1.5 T	1^st^ gen. 3 T	2^nd^ gen. 3 T
TR/TE [ms]	6300/79	6600/80	6000/68
Sequence type	EPI-SE		
FOV [mm×mm]	380×297		
Matrix	192×150		
Slice thickness [mm]	6		
Interslice gap [mm]	0		
Spatial resolution [mm^3^]	2.0×2.0×6.0		
Number slices	35		
b-values	0, 50, 100, 200, 400, 800		
Parallel imaging	GRAPPA 2		
Acquisition time [min]	7:02	7:22	6:54
Respiratory control	Free breathing		
Fat suppression	SPAIR		
Averages	4		
Bandwidth [Hz/px]	1628		

Volunteers underwent DWI in all three of the scanners in a random order within the same day with no more than 10 minutes between each of the examinations.

### Image Analysis

**Figure 1:**
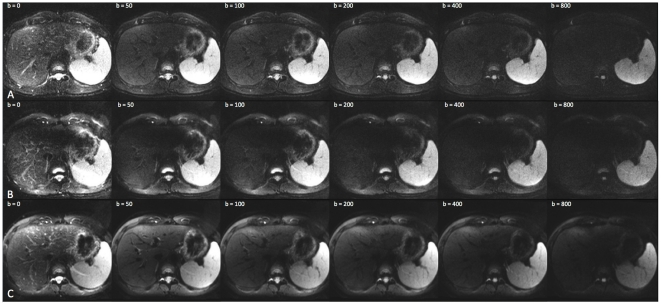
Representative ADC-images. Representative ADC-images positioned at the same level of the left lobe of the liver from the same volunteer in all three scanners (A–C). Inhomogeneous signal is seen particularly in the left lobe of the liver with the first generation 3 T scanner (B).

For each patient and volunteer the system-generated ADC parameter maps were used for further analysis. The three MR systems used the same mono-exponential fitting algorithm with a noise level in the automatic ADC-map generation which was kept constant at 10 for all examinations. In each ADC parameter map, ROIs were selected manually over seven anatomical distributions, which were chosen mostly due to clinical significance or had been recognized to suffer image degradation in the first generation 3 T systems: right lobe of the liver, left lobe of the liver, caudate lobe of the liver, head of the pancreas, right kidney, left kidney, and spleen. The ROIs were placed by a radiologist who was blinded to the MR-system used. Reasonable care was taken to measure only the intended region without contacting structural borders or obvious vasculature within the anatomical segment. The mean signal intensity of the ROI was used as the ADC value for further analysis. The average size of the ROI selected was 1.5 cm^2^ (Figure 1). For the ROI analysis an OsiriX DICOM viewer (OsiriX 3.7.1; The OsiriX Foundation; Geneva, Switzerland) running on a commercially-available MacPro (Apple, Cupertino, CA) was used. This procedure was repeated with all 150 of the clinical patients and in all 57 (19 volunteers with 3 studies each) of the volunteer studies.

### Statistical Analysis

Statistical analysis was performed using JMP 9.0 (SAS Institute, Cary, North Carolina, USA). Continuous variables are expressed as mean ± standard deviation (SD). The Shapiro-Wilk test was applied to determine the probability distribution. Comparisons of normally distributed tests within the patient and volunteer groups were performed with analysis of variance (ANOVA) and post hoc analysis with t-tests for independent samples. Within the patient and volunteer groups, data that were not normally distributed were examined with the Kruskal-Wallis test and post hoc Mann-Whitney U test with Bonferroni correction. Depending on normality of data distribution, Pearson or Spearman rank correlation coefficients were determined to investigate the correlation between ADC values from the three different MRI scanners. Limits of agreement between the three different MR scanners were calculated with Bland-Altman analyses showing the mean value of difference of each pair plotted against the average value of each pair. A two-tailed p-value of <0.05 was considered statistically significant. As this study was designed as an exploratory study no sample size estimation was performed beforehand.

## Results

**Figure 2:**
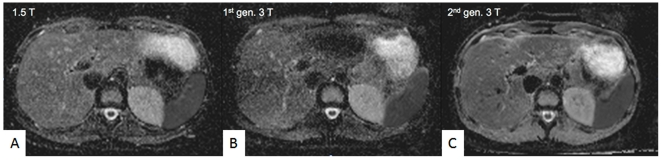
Representative source data from all three MR-scanners. Representative source data images taken from the 1.5 T MR scanner (A), the first generation 3 T MR-scanner (B) and the second-generation 3 T MR-scanner (C) show that the second-generation 3 T MR-scanner yields higher signal to noise ratio throughout all b-values which is particularly well appreciated at the higher b-values.

MR imaging was successfully completed once in all 150 patients and three times each of the 19 volunteer studies. Mean ADC values were calculated in each of the seven anatomical distributions for both populations; the collected data are depicted in Tables 3 and 4. Exemplary images from the volunteer studies are shown in Figure 1 (ADC-values) and Figure 2 (DWI source data).

**Table 3:** Mean ADC values (×10^−3^ mm^2^/s) in the patient population.

	1.5 T	1^st^ gen. 3 T	2^nd^ gen. 3 T
**Pancreas (head)**	1.20±0.30	**0.94±0.55**	1.22±0.30
**Liver (left lobe)**	1.09±0.14	**0.35±0.24**	1.11±0.16
**Liver (caudate lobe)**	0.91±0.20	**0.44±0.41**	0.96±0.19
**Liver (right lobe)**	0.97±0.15	**0.57±0.37**	0.95±0.12
**Right Kidney**	1.84±0.39	**1.69±0.77**	1.82±0.38
**Left Kidney**	1.89±0.53	**1.77±1.10**	1.91±0.55
**Spleen**	0.83±0.23	**0.73±0.36**	0.79±0.18

ADC values in bold are significantly different (p<0.05) from the other two values in the same distribution. There are no significant differences (p≥0.05) between mean ADC values between two values that are not bolded. The deeper regions, which are more susceptible to B_1_ inhomogeneity artifacts, are listed in rows 1–3.

**Table 4:** Mean ADC values (×10^−3^ mm^2^/s) in the volunteer population.

	1.5 T	1^st^ gen. 3 T	2^nd^ gen. 3 T
**Pancreas (head)**	1.27±0.22	**1.07±0.29**	1.32±0.21
**Liver (left lobe)**	0.99±0.25	**0.32±0.20**	1.00±0.27
**Liver (caudate lobe)**	0.82±0.29	0.62±0.35	0.84±0.30
**Liver (right lobe)**	0.98*±0.23	0.76*±0.27	0.84±0.25
**Right Kidney**	1.86±0.44	1.81±0.44	1.83±0.44
**Left Kidney**	1.91±0.11	1.92±0.11	1.90±0.14
**Spleen**	0.83*±0.08	0.82±0.11	0.76*±0.08

ADC values in bold are significantly different (p<0.05) from the other two values in the same distribution. ADC values with an asterisk are significantly different from each other. There are no significant differences (p≥0.05) between mean ADC values between two values that are not bolded or have an asterisk. The deeper regions, which are susceptible to B_1_ inhomogeneity artifacts, are listed in rows 1–3.

In the patient population, the mean ADC values in both the peripheral and central distributions illustrate the same pattern: the values between the first generation 3 T were significantly different from the other two systems (p<0.01) and tended to be lower. The differences between the first generation 3 T and the other two MR scanners ranged from roughly 5% (in the kidneys) up to 200% (in the left lobe of the liver). The ADC values measured between the 1.5 T and second generation 3 T systems were nearly identical, with no significant differences between the two systems (p>0.05).

**Figure 3:**
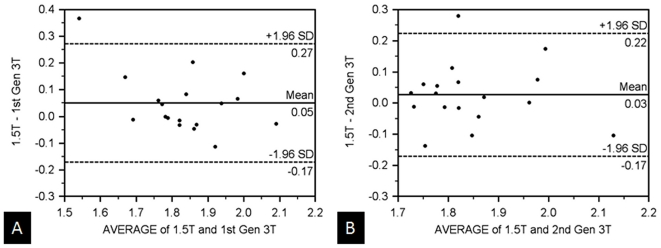
Comparison of renal ADC-values between all three MR-scanners. Bland-Altman plot of mean ADC values from the right kidney comparing the 1.5 T system with the (A) first generation 3 T and (B) second generation 3 T MR-scanners in the volunteer population. The mean ADC values are similar amongst all three systems. (x-axis: average/y-axis: difference of ADC at 1.5 T and the corresponding 3 T systems (×10-3 mm^3^/s)).

In the volunteer population, although the same general trend of similar ADC measurements between the 1.5 T and second generation 3 T existed, the differences between these systems and the first generation 3 T were not significant in all the regions. In the kidneys, the mean ADC values were similar amongst all three scanners with no statistical differences in mean ADC measurements (1.86×10^−3^ mm^2^/s, 1.81×10^−3^ mm^2^/s, 1.83×10^−3^ mm^2^/s, with p>0.05). Table 5 depicts the correlation coefficient (r-values) in these regions and shows the correlation strength between the 1.5 T scanner and the first generation 3 T scanner to be slightly weaker than the differences between the 1.5 T scanner and the second generation 3 T scanner (r = 0.51–0.72 versus 0.64–0.88, respectively). A Bland-Altman plot of the volunteers' ADC values from the kidneys comparing both 3 T systems to the 1.5 T scanner (Figure 3) illustrates similar values with minimal mean differences (<0.05×10^−3^ mm^2^/s) and a relatively small standard deviation of these differences. The mean ADC values involving the right lobe of the liver appeared less similar across all three scanners with statistically different measurements between the 1.5 T and first generation 3 T systems (p = 0.02); the differences of the 1.5 and second generation 3 T scanner were not significant. Although the mean ADC values were similar in the spleen, the correlation strength was low (r = 0.18–0.62).

**Table 5:** Correlation coefficients (r) of mean ADC values between the three systems.

	1.5 T vs 1^st^ gen. 3 T	1.5 T vs 2^nd^ gen. 3 T	1^st^ gen. 3 T vs 2^nd^ gen. 3 T
**Pancreas (head)**	0.66	**0.92**	0.66
**Liver (left lobe)**	0.41	**0.92**	0.35
**Liver (caudate lobe)**	**0.91**	**0.90**	**0.77**
**Liver (right lobe)**	**0.87**	**0.85**	**0.89**
**Right Kidney**	**0.72**	0.64	0.63
**Left Kidney**	0.51	**0.88**	0.59
**Spleen**	0.62	*0.18*	*0.22*

Values of r>0.7 are bolded to illustrate strong correlation, while values that have low correlation (<0.5) are italicized. The deeper regions, which are susceptible to B_1_ inhomogeneity artifacts, are shaded in gray.

**Figure 4:**
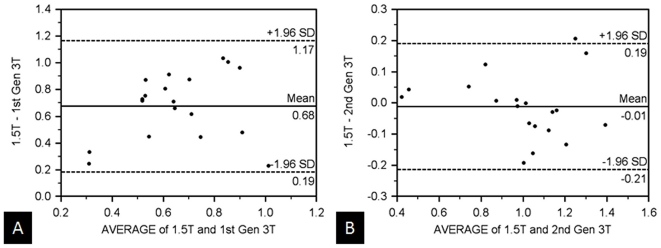
Comparison of hepatic ADC-values between all three MR-scanners. Bland-Altman plot of mean ADC values from the left lobe of the liver comparing the 1.5 T system with the (A) first generation 3 T and (B) second generation 3 T scanners in volunteers. Mean ADC values of the first generation 3 T are on average 0.68×10^−3^ mm^2^/s lower than those measured on the 1.5 T system. Mean ADC values are similar in the 1.5 T and second generation 3 T scanners. (x-axis: average/y-axis: difference of ADC at 1.5 T and the corresponding 3 T systems (×10-3 mm^3^/s)).

**Figure 5:**
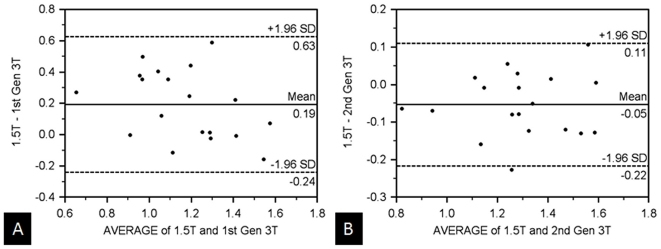
Comparison of pancreatic ADC-values between all three MR-scanners. Bland-Altman plot of mean ADC values from the head of the pancreas comparing the 1.5 T system with the (a) first generation 3 T and (b) second generation 3 T scanners in voluteers. Mean ADC values of the first generation 3 T are on average 0.19×10^−3^ mm^2^/s lower than those measured on the 1.5 T system. Mean ADC values are similar in the 1.5 T and second generation 3 T scanners. (x-axis: average/y-axis: difference of ADC at 1.5 T and the corresponding 3 T systems (×10-3 mm^3^/s)).

In the left lobe of the liver and the head of the pancreas, although there were significantly lower ADC values between the first generation 3 T and the other two scanners (p<0.05), there were no significant differences in mean ADC values between the 1.5 T and second generation 3 T systems (left lobe of the liver: 0.99×10^−3^ mm^2^/s vs 1.00×10^−3^ mm^2^/s; and head of the pancreas: 1.27×10^−3^ mm^2^/s vs 1.32×10^−3^ mm^2^/s, respectively, in which r = 0.92 for both regions). Bland-Altman plots of the deeper and centrally-located left lobe of the liver and head of the pancreas (Figures 4 and 5) depict this information by showing nearly no differences in the mean ADC values ( = 0.01×10^−3^ mm^2^/s) and a narrower standard deviation of the differences. In the aforementioned regions, comparison of the 1.5 T and first generation 3 T scanners revealed lower mean ADC values from the first generation 3 T system than those measured at 1.5 T (e.g. by 0.68×10^−3^ mm^2^/s in the left lobe of the liver and by 0.19×10^−3^ mm^2^/s in the head of the pancreas). The correlation coefficient comparing the two systems was low in the left lobe of the liver (r = 0.41). The mean ADC values in the caudate lobe were similar between the 1.5 T and second generation 3 T (0.82×10^−3^ mm^2^/s and 0.84×10^−3^ mm^2^/s, with r = 0.90); mean ADC values obtained from the first generation 3 T scanner were lower (0.62×10^−3^ mm^2^/s).

## Discussion

An appealing feature of quantitative radiology is the ability to obtain absolute measures of tissue characteristics which can be applied for detection of disease or longitudinal monitoring of disease resolution/progression. However, the findings of this study clearly demonstrate that quantitative radiology by measuring ADC is heavily dependent on the MR-equipment used. In the patient and volunteer populations, differences in ADC of up to 200% were seen between the first generation 3 T MR system and the other two systems. The biggest differences in ADC were encountered in the liver, an organ which is well known for harboring metastases and hence of high oncologic interest. In the liver, the first generation 3 T system almost always yielded lower ADC results than the second generation 3 T MR system as well as the 1.5 T MR system, the latter of which can be considered as the current clinical standard of reference. While the differences were significant throughout all anatomical regions in the patient group, the relatively small number of volunteers might be the cause for some of the non-significant differences in the volunteer group. Another factor that might have led to the more pronounced difference in ADC measurements between the patient and the volunteer groups is the fact that only healthy volunteers were included, while several patients had anasarca or ascites. Both conditions might negatively influence DWI signal intensity, and thus also change the ADC value. The lower ADC values with the first generation 3 T MR system can probably be attributed to imperfect RF-excitation and B_1_ inhomogeneities. At 1.5 T the RF-excitation wavelength is roughly 70 cm while it is 35 cm at 3 T [15]. Due to this short wavelength at 3 T, constructive and destructive interferences occur, with the latter leading to local signal decay and even potentially complete signal loss. Anatomical regions prone to this are the left lobe of the liver, the caudate lobe of the liver, and the head of the pancreas (which were grouped as central regions in this study). The introduction of parallel transmission techniques seems to substantially overcome the limitation otherwise experienced in 3 T, as seen by the almost identical ADC values between the 1.5 T and second generation 3 T MR systems. As other external limitations, such as the four-fold higher susceptibility experienced at 3 T remain unchanged, the observed difference in ADC in the intraindividual volunteer study can probably be attributed to the parallel transmission. Without parallel transmission the poor signal intensity encountered, particularly in the diffusion weighted source data images with b = 400 s/mm^2^ and b = 800 s/mm^2^, led to an erroneously low calculation of the ADC in the first generation 3 T MR scanner. In a previous similar study which compared the 1.5 T MR-system with a first generation 3 T MR-system a significantly lower image quality was found for the 3 T MR system in 8 volunteers [16]. In this study the differences in ADC between the 1–5 T MR-system and the first generation 3 T MR-system were statistically not significant but problematic regions such as the head of the pancreas and the left lobe of the liver were simply omitted. In our study, the difference in ADC was least pronounced in the kidneys and the spleen, with differences in the patient population ranging from 5% to 10% between the first generation 3 T and the other two MR systems. Although these organs are located peripherally and less often affected by standing wave artifacts, they are highly vascular and the laminar flow from smaller vessels and capillaries contribute to a higher ADC calculation (particularly at lower b-values); this higher contribution of signal from sources which are more variable, rather than simply restricted diffusion of water particles, may have led a to lower correlation strength of the mean ADC values taken between the 1.5 T and second generation 3 T in the spleen and kidneys. These findings can potentially be explained by the intravoxel incoherent motion (IVIM) theory [17]. The relationship of the IVIM model to the ADC means/correlative strength across the three scanners are outside the scope of this study and will therefore be investigated separately.

Initial evaluations of DWI within the abdomen from newer second generation 3 T systems from a different vendor which incorporate another dual-source RF excitation technique were promising as they suggested improved image quality when compared to their first generation counterparts [13], [14]. The impact of field strength and excitation technique on measured ADC values was not assessed in either of these two studies. Although ADC values are poorly transferable between 1.5 T and first generation 3 T systems [12], there is a clinical need to identify whether ADC values obtained between 1.5 T and newer 3 T systems are transferable, as more hospitals are acquiring higher field strength MR systems and the ability to obtain consistent ADC values within these systems can influence clinical decisions, particularly in oncologic imaging. Currently measurements of tumor response to antineoplastic agents are mainly performed either under a set of published guidelines referred to as Response Evaluation Criteria in Solid Tumors (RECIST) or the WHO guidelines, both of which involve some combination of size measurements of primary lesions and lymph nodes. Potential pitfalls in this approach include the delay or lack of change in size of the lesion, even when tumor vascularity may already decrease. Such cases could falsely be noted as late or nonresponders to chemotherapeutic agents. However data suggests that echo planar imaging with DW sequences can serve to predict early response in primary and metastatic malignancies located in the liver, small and large bowel, and pelvic organs as early as weeks to days after treatment—well before there is a reduction in lesion size [18], [19], [20], [21], [22]. Given the cost and adverse effects of many antineoplastic drugs, earlier detection of response may prevent overuse. There has even been suggestion that DWI with ADC measurements can be used to assess effectiveness of antineoplastic drugs in Phase I/II clinical trials [5], [6].

Early data in the detection and identification of malignant lesions has also been encouraging, although with limitations. Numerous investigators have attempted to uncover ADC threshold values to distinguish malignant from benign lesions. However in different studies, investigators have suggested an inconsistent set of ADC threshold values to make this distinction. For example, regarding renal malignancies, ADC threshold values have ranged from 1.15 to 2.4×10^−3^ mm^2^/s [23], [24], [25], [26], [27]. In a recent meta-analysis of ADC values of hepatic tumors, Li et al concluded that although ADC values were useful for differentiation of liver lesions, the marked heterogeneity between the pooled studies limited a universal threshold ADC value [28]. Numerous variables cause inconsistent data, including the number and value of chosen b-factors, imaging parameters (including TR, TE, slice thickness), and scanning techniques (breath hold versus respiratory-triggered). To make matters more complex, although most investigations have been performed on 1.5 T systems, 3 T scanners are becoming more ubiquitous. In aims to use DWI as a cancer biomarker and recommend methods to make more reproducible ADC values, a panel of 100 experts mentioned the inherent limitations of traditional (first generation) 3 T systems [6]. For this reason, when acquiring ADC data within the abdomen, the more commonly used field strength has and would probably continue to be 1.5 T. Nonetheless, this has not curbed the rate that 3 T MR systems continue to be acquired by hospitals and practices. We therefore believe that by presenting consistent measurements between 1.5 T and second generation 3 T systems, our study provides useful data that may eventually influence how DW images can be obtained within the abdomen.

### Study Limitations

This study has some limitations. For one, three averages were used when acquiring DW images on a second generation 3 T, compared to 4 of the other two systems in the patient group. The different values could cause a smaller signal average in the second generation 3 T system which might lead to a slightly lower ADC calculation than would be expected with a higher signal average. The same holds true for the thinner slice thickness used at 3 T in comparison to 1.5 T. To address these potential pitfalls the volunteer study was performed with identical sequence parameters as far as possible. Besides this, different b-values were used in the patient and volunteer populations. The patient population used three b-values (50/400/800 s/mm^2^), two-thirds of which were higher than 200 s/mm^2^, while the volunteer population used more b-values (0/50/100/200/400/800 s/mm^2^), of which only one-third were higher than 200 s/mm^2^. The selection of b-values in the patient population was based on the vendor's default setting for abdominal DWI. The higher proportion of b-values chosen less than 200 s/mm^2^ actually weighted diffusion signals contributed by capillary perfusion more heavily in the volunteer population. This may contribute to some of the more heterogeneous findings identified in the spleen and kidneys, and as described before can possibly be explained by the IVIM theory. Despite this potential bias, this approach was used as ADC calculations based on a larger set of b-values becomes clinically more important. Also, future studies might include even more than 6 b-values to reliably establish normative values for IVIM-ADC. Due to time constrains this study did not investigate further into this direction. Further factors that may also affect the ADV values such as the strength of the gradient pulse and the time interval between gradient pulses might have influenced the results, too. By using the standard sequences provided by the vendor this confounding factor has been minimized. Of course, our results are technically confined to the MR-systems included in this study. A generalization to MR-systems from other vendors cannot safely be assumed. Finally, the small sample size of volunteers might explain the lacking significance in in some of the distributions.

In conclusion, these data suggest that in clinically-relevant regions, nearly identical values with a high correlation can be obtained between 1.5 T and 3 T systems which implement dual-source parallel RF excitation techniques. Although this study focused on normal (nonpathological) anatomy, the early findings may suggest that if all other variables are controlled, threshold ADC values may eventually be used interchangeably between scanners obtaining DW images at different field strengths.
